# Nutrition and Physical Activity in an Interdisciplinary Approach to Migraine: A Narrative Review

**DOI:** 10.3390/nu17243869

**Published:** 2025-12-11

**Authors:** Roberto Pippi, Deborah Prete, Marco Alabiso, Paola Sarchielli

**Affiliations:** 1 Healthy Lifestyle Institute, Centro Universitario Ricerca Interdipartimentale Attività Motoria (C.U.R.I.A.Mo.), Department of Medicine and Surgery, University of Perugia, Via G. Bambagioni, 19, 06126 Perugia, Italy; deborah.prete@collaboratori.unipg.it; 2 Section of Neurology, Department of Medicine and Surgery, University of Perugia, Piazzale Gambuli, 1, 06129 Perugia, Italy; marcoalabiso.1995@gmail.com (M.A.); paola.sarchielli@unipg.it (P.S.)

**Keywords:** migraine, healthy diet, lifestyle, physical activity, obesity, supplements

## Abstract

**Background/Objectives**: Migraine (MIG) is a neurologic, acute or chronic, disabling pathology that significantly reduces quality of life in millions of people worldwide. Among modifiable factors that influence the onset and management of MIG, nutritional and physical activity habits are crucial elements of a non-pharmacological treatment aiming at improving the anti-inflammatory condition. **Methods**: This review analyses the evidence available, using the last 10 years of published papers (searching in MEDLINE/PubMed), on the use of specific dietetic plans, the identification of potential nutritional triggers, the role of some supplements, the effects of regular PA, and weight management, in people with MIG. **Results**: Associations have been reported between the use of ketogenic, low-glycemic, and anti-inflammatory dietary patterns, the identification of potential nutritional triggers, and supplementation with some elements such as any vitamins, PUFAs, and CoQ10, in addition to regular mixed PA, and the duration, frequency, and intensity of MIG attacks. **Conclusions**: Despite some RCTs showing promising results, an actual lifestyle-based protocol does not yet exist due to methodological limitations. However, current evidence supports the development of a “lifestyle” approach to MIG management, although further research is needed to establish definitive and standardized clinical recommendations.

## 1. Introduction

Migraine (MIG) is a “a common disabling primary headache disorder” [1,2] characterized by repeated attacks of headache of moderate or severe intensity, pulsating in quality and aggravated by routine physical activity (PA) which is accompanied by discomfortable symptoms (such as nausea or vomiting, photo- and phonophobia, kinesiophobia, etc.), significantly reducing patients’ quality of life [3,4]. It is estimated that over a billion people are currently affected worldwide [5]. MIG was classified as among the leading causes of disability worldwide, and the annual prevalence was 14.2%, according to the Global Burden of Disease study (2021) [6].

Given the complex nature of MIG, it is not easy to distinguish between risk factors, triggers, and clinical outcomes. Therefore, adequate information on the causes and aggravating factors is essential to manage both the rate and seriousness of attacks. In addition, specific non-pharmacological interventions, such as PA (i.e., mixed exercise intervention) and anti-inflammatory dietary patterns (i.e., Mediterranean diet, or MD [7,8,9], a low glycemic index diet [8,10,11], ketogenic diet, or KD), could reduce the duration, number of MIG attacks, and pain intensity.

The literature [12,13] on managing and treating MIG supports a multidisciplinary approach that includes both acute and preventive pharmacological treatment, evidence-based behavioral interventions, and lifestyle modification [14,15]. Moreover, weight loss strategies may be beneficial in improving conditions related to MIG, particularly in individuals with obesity [15]. Monitoring, regulating, and improving sleep, stressors, PA, and nutritional habits (NHs) can be linked to the frequency and severity of attacks, improving patients’ quality of life [16,17]. For this, recently, Robblee and Starling suggested the acronym “SEED”, which stands for Sleep, Exercise (EXE), Eat, and Diary, which could be helpful to remind us of the lifestyle habits to focus on to manage MIG attacks [18].

**Figure 1:**
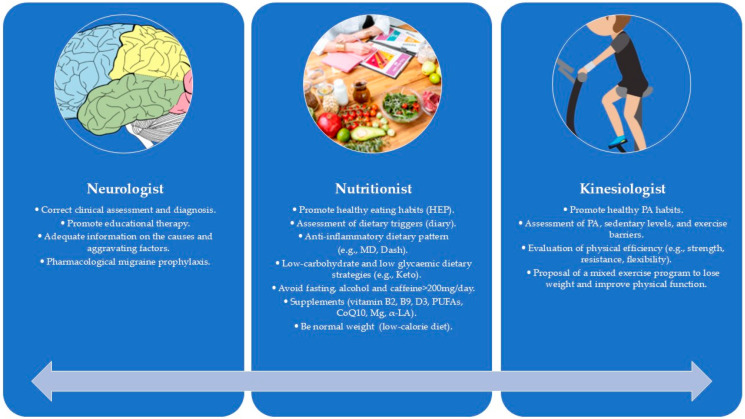
The interdisciplinary approach to MIG.

Some scientific societies, such as the American Headache Society [14], the French Headache Society [19], the Danish Headache Society [20], and the European Federation of Neurological Societies [21], promote the adoption of non-invasive interventions to support drug therapy, such as nutritional and PA individualized approaches (Figure 1).

Considering the impact of this disease on the world population, it is crucial to elicit all the non-pharmacological strategies to counteract MIG-related comorbidities. In this narrative review, we aim to revise the importance of diet, nutritional triggers and supplements, PA, and weight management in the prevention and treatment of MIG in the adult population. As a narrative review, the article cannot draw causal conclusions nor support clinical recommendations, and the findings should be interpreted accordingly.

## 2. Materials and Methods

### 2.1. Search Strategy

For this review, we searched studies utilizing the MEDLINE (PubMed) database using the following search strings: “physical activity and migraine” OR “nutrition and migraine” OR “diet and migraine” OR “supplements and migraine” and reviewing each title and abstract of the papers. A database search was conducted from September 2024 to May 2025, considering only papers published in English, within the last ten years. The digital research was independently conducted by two of the authors (R.P., D.P.).

### 2.2. Study Selection

**Figure 2:**
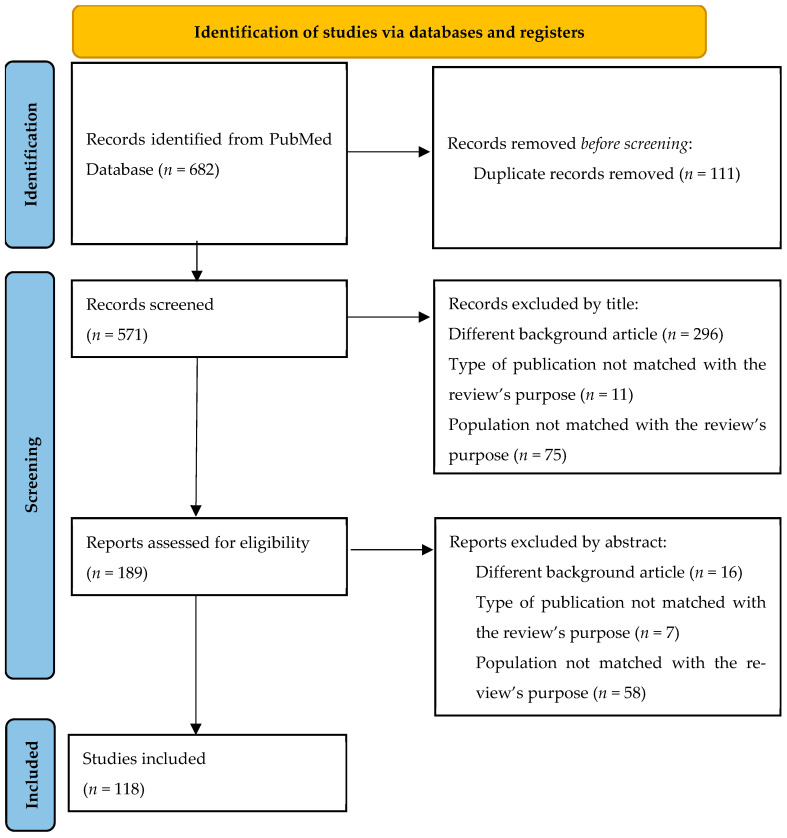
Flow diagram showing the included studies.

Studies must meet the following requirements to be eligible: systematic reviews, reviews, meta-analyses, retrospective or prospective observational studies, randomized controlled trials (RCTs), practice guidelines studies with adult patients with long-term conditions (that are not psychiatric), undergoing any dietary and/or physical activity (non-rehabilitative therapies) interventions. Exclusion criteria were studies involving animals, patients aged ≤18 and >65 years; editorials, letters, case reports, book chapters, or comments; papers published in any language other than English. The search initially produced 682 results. Articles were selected by reading the titles and the abstracts. Then, we excluded duplicates using Rayyan, a web application [22] available at link http://rayyan.qcri.org (accessed on 8 December 2025), according to the flow diagram presented in Figure 2.

## 3. Results

### 3.1. Pathophysiological Theories

The pathophysiology of MIG was primarily attributed to neurological and vascular mechanisms [23]. Neurotransmitters, such as serotonin, dopamine, and glutamate (the primary excitatory neurotransmitter in the brain), play a vital role in MIG development and treatment. For instance, drug therapies target serotonin receptors in the brain and cranial blood vessels to enhance serotonin signaling, leading to pain relief through blood vessel constriction and the inhibition of specific neuropeptides, like substance P and calcitonin gene-related peptide (CGRP) [24]. Some studies have explored pathophysiological mechanisms which involve cortical spreading depression (CSD) and direct stimulation of meningeal nociceptors, and hyperexcitability of the trigeminovascular system. They seem to linked to impaired mitochondrial energy production, resulting in an increased production of reactive oxygen and nitrogen species (ROS) beyond physiological levels. Mitochondrial dysfunction or energy deficiency in the brain appears to underlie the pathogenesis of MIG and it could be linked to the impact of MIG attacks [25]. Furthermore, certain research efforts have investigated how the activation of the trigeminovascular system initiates a neurogenic inflammatory response, leading to the release of pro-inflammatory neuropeptides such as CGRP and substance P.

Recent research has identified CGRP, pro-inflammatory agents such as monocyte chemoattractant protein-1 (MCP-1), and cytokines like tumor necrosis factor (TNF)-α, interleukin-1β (IL-1β), and interleukin-6 (IL-6) as contributors to MIG pathogenesis. Musubire et al. [26] reported that IL-6, TNF-α, and IL-8 are higher in patients with MIG than in healthy controls.

Recent studies [27,28] suggest that a deficiency in the endocannabinoid system (ECS), a neuro-modulatory system that influences pain processing and modulation, may be a significant factor contributing to MIG pain and play a leading role in MIG persistence.

Moreover, the opioid system is another structure involved in MIG pain [29]. An endogenous opioid, the beta-endorphin (BE) neurotransmitters, are involved in modulating nociceptive circuits. Moreover, BE has been shown to have a more potent analgesic effect than morphine [30].

Recently, however, it has become clear that metabolic factors play a significant role in this disorder [23]. MIG triggers often relate to metabolic imbalances, including fasting, changes in sleep patterns, hormonal shifts, EXE, alcohol, and sodium chloride (NaCl) consumption, and fluctuations in weather. Stress, sedentary lifestyle, and malnutrition (due to deficiency or excess) can increase chronic inflammation [31]. Although the evidence remains preliminary, it seems that inflammation and elevated levels of substance P could lead to the widening of arteries and headaches, which are the most typical symptoms of an MIG attack. Some authors, proposing a scoring system, hypothesized that ischemic stroke risk in MIG patients it is predictable considering factors like hypertension (related also to the high NaCl intake), diabetes, high BMI, and atrial fibrillation, along with MIG characteristics and certain medications, regardless of aura presence. These tools, along with the identification of MIG causes, could help clinicians to screen high-risk MIG patients and to suppose preventive strategies [32], with an interdisciplinary approach.

### 3.2. Relationship Between DIET and MIG

Diet has been observed to influence the modulation of neuroreceptors, neuropeptides, and sympathetic nerve responses and may therefore be connected to both the development and relief of inflammation. Adhering to anti-inflammatory dietary tips, such as those recommended by the National Headache Foundation (NHF) [33], or increasing the intake of anti-inflammatory foods and nutrients (for example, some authors suggested the Omega-3polyunsaturated fatty acids (PUFAs) importance [34,35]), may help to manage the duration, frequency, and severity of MIG attacks. Associations have been reported between PUFAs and inflammation balance, antinociceptive, antioxidant, and neuromodulatory features that could be effects to the cardiovascular and nervous systems. Moreover, it seems that they stimulate the generation of specialized pro-resolving mediators (SPMs), which support neuroinflammatory resolution and pain modulation by facilitating communication at the glia-neuron interface, potentially contributing substantially to MIG symptom relief [36]. Specific dietary protocols may contribute to MIG management by reducing the intake of sensitizing substances known to enhance central sensitization and promote headache chronicity (Table 1). A variety of dietary components—including alcohol, caffeine, chocolate, monosodium glutamate (MSG), nitrates, and tyramine—have been consistently reported as common MIG triggers [9]. In particular, large amounts of aspartame and MSG can trigger an MIG attack [37]. In this context, it is reasonable to investigate whether specific nutrients, particularly those engaged in metabolic energy processes and reactive oxygen species (ROS) stress regulation [6,38], could support pharmacological treatment in the prophylaxis and management of MIG.

In susceptible individuals, specific dietary patterns may induce MIG attacks, potentially through neuroendocrine pathways [39]. A key mechanistic link between nutrition and MIG appears to involve impaired glucose metabolism [9]. Hypoglycemia, for instance, has been shown to prolong CSD, a phenomenon strongly associated with MIG pathogenesis [26]. Nutritional ketosis, which provides ketone bodies as alternative energy substrates, may mitigate hypoglycemia and thereby reduce the incidence and intensity of CSD [40].

Low-carbohydrate dietary strategies (for example, the Ketogenic Diet (KD), Modified Atkins Diet (MAD), and Very-Low-Calorie Ketogenic Diet (VLCKD)), have garnered increasing attention in this context. These diets could influence MIG-related pathways through the neuromodulator effects of ketone bodies, which act as signaling molecules with established roles in neuroprotection, mitochondrial function, and cellular energy homeostasis. In addition to improving metabolic efficiency, such diets may exert anti-inflammatory effects by compensating for serotonergic dysfunction, reducing CGRP levels, downregulating pro-inflammatory cytokines, and attenuating microglial activation [41,42,43]. Collectively, these mechanisms may contribute to the attenuation of both acute and chronic MIG symptoms. According to a 2025 review, scientific interest has also focused on ketogenic diets and low-fat diets enriched with omega-3 fatty acids exploring their potential role in the management of MIG in relation to neuroprotective and anti-inflammatory hypotheses. These approaches are discussed in relation to their potential to influence on mitochondrial function and vascular tone, with possible implications for the modulation of oxidative stress and calcitonin gene-related peptides which are often mentioned in pathophysiological mechanisms of MIG [44]. Emerging evidence also highlights the relevance of the gut–brain axis in MIG pathophysiology [45,46]. Although the precise mechanisms remain incompletely understood, an increase in intestinal permeability has been hypothesized as a possible factor to provide an overview of different diet approaches explored in MIG. The impact of diet on preventing and treating MIG is now widely discussed, but there seems to be no valid evidence to support a specific single nutritional strategy [17]. However, it is now known that implementing good habits, especially a healthy diet, and following weight loss approaches for comorbid obesity can have beneficial effects [18,41,47,48]. One strategy could be the Healthy Eating Plate (HEP), developed by Harvard University [49], which was highlighted in a 2020 interventional study as comprehensive dietary model designed to promote balanced nutrition and support overall health outcomes [50]. However, the HEP diet takes longer to achieve its beneficial effects. A cross-sectional study conducted by S. Hajjarzadeh, using a 168-item food frequency questionnaire (semi quantitative), examined the association between adherence to certain eating habits- including the consumption of fish, fruits and vegetables—and the frequency of MIG episodes [51]. According to the SEEDS study [18], there is no single, diet for MIG; instead, you should eat healthy meals at least three times a day, preserve adeguate hydration, avoid fasting, limit caffeine intake, and maintain a normal weight (BMI 18.5–25).

Fasting has been associated with MIG attacks [41], particularly when breakfast is skipped [52]. Although limited, available research suggests that hydration status may play a protective role [18]. A cross-sectional study [53] observed a possible link between daily water consumption and reduced headache frequency and duration. According to a 2020 review [54], alcohol and caffeine are among the most frequently reported dietary triggers associated with increased MIG frequency. The effects of caffeine appear to be dose-dependent: low quantities may be associated with antioxidant and anti-inflammatory effects, whereas higher intake could act as a potential trigger. A 2023 scientific report noted a possible link between dietary caffeine intake and the occurrence of severe MIG in adults. Specifically, each 100 mg/day increase in caffeine consumption was associated with a 5% higher prevalence of these conditions. Moreover, individuals consuming ≥400 mg/day exhibited a 42% higher prevalence compared to those with an intake between 0 and <40 mg/day [55]. Suggested practices advise limiting caffeine consumption to less than 200 mg/day, possibly because both caffeine and theine contain thiaminase. This enzyme degrades thiamine, a compound considered protective in MIG pathophysiology [26]. However, thiamine also plays a positive role in mitochondrial function, particularly within the electron transport chain at complex IV. Alcoholic beverages, particularly red wine, are commonly reported by individuals with MIG as potential triggers. Alcohol may affect renal regulation of serum sodium (Na), which has been observed to be elevated in MIG patients, particularly during an attack [37]. The risk of MIG increases notably with the consumption of five or more servings of alcohol per day [56,57]. Alcohol is thought to contribute to oxidative stress through the production of reactive ROS. Interestingly, in habitual consumers, sudden abstinence from alcohol may itself become an MIG trigger.

Elimination diet strategies have been employed to identify and reduce potential dietary triggers of MIG attacks, but their overall success has been limited. Although numerous foods have been proposed as possible triggers, strong evidence currently exists only for alcohol and caffeine [17]. Nevertheless, some researchers, including Alpay, Bunner, and Özön [39], have examined elimination diets targeting foods associated (e.g., nuts and seeds, spices, cheeses, seafood, sweetened foods, fruits, and vegetables) with elevated IgG antibody levels. These authors reported a reduction in mean attack count, frequency, and pain intensity. To ensure nutritional adequacy, elimination of diets should be personalized and guided by a registered dietitian [48]. Personalized dietary interventions, combined with patient education and maintaining a headache diary, may help individuals identify and avoid potential triggers while promoting the adoption of sustainable and health-promoting dietary patterns [6].

The risk of MIG appears to be higher among individuals adhering to a Western dietary pattern, characterized by the consumption of processed meats, fast food, sweetened foods, snacks, sugary beverages, fruit juices, refined grains, and high NaCl intake [58]. Conversely, individuals who consume greater amounts of vegetables, fruits, legumes, fish, and low fat dairy products seem to show a reduced risk of developing MIG [9,59]. The level of dietary antioxidant compounds -including vitamins A, E, and C, carotenoids (found in plant foods), selenium, and zinc- has been observed to be associated with the occurrence of severe headaches or MIG [60]. A cross-sectional study [9] found that adherence to the Mediterranean diet is associated with a lower frequency and duration of headache episodes, as well as reduced scores on the Migraine Headache Index Score (MHIS) and the Headache Impact Test-6 (HIT-6). Similarly, a recent study [11] demonstrated that an individualized low-glycemic diet (LGD)—defined as the intake of 60–80 g/day of carbohydrates from low-glycemic-index sources—may serve as a practical non-pharmacological approach to MIG prophylaxis. Another study involving 294 patients reported that following a low-glycemic diet for three months significantly reduced the frequency and severity of MIG attacks when compared to conventional pharmacological treatments [8]. Some Italian researchers [61] have explored the correlation between higher consumption of whole foods, rather than refined products, and the frequency of MIG attacks, as well as reliance on medication. Additional dietary interventions, such as the Dietary Approaches to Stop Hypertension (DASH) and the Ketogenic Diet (KD), have been associated with changes in the frequency, duration, and intensity of MIG episodes in adults [48,62]. Interventions like the DASH diet may be linked to lower MIG occurrence, potentially through effects on blood pressure via limited NaCl intake [7,41,58], particularly MSG, which has been reported in relation to the onset of headaches. According to Obayashi et al. [63], MSG in high concentrations (>2%) in solution (as opposed to amounts typically consumed in food) has been shown to induce headaches. According to Brown RB, there appears to be a relationship with salt intake, especially due to high consumption of processed foods, which may contribute to fluid retention. This can result in a condition similar to the water retention observed prior to an MIG attack. Since some reported warning signs include craving salty foods and increased thirst, reducing NaCl intake has been suggested as a potential strategy to manage MIG symptoms. In individual with high blood pressure who use nonsteroidal anti-inflammatory drugs (NSAIDs), these medications may relieve pain but have also been associated with increased Na retention, which can complicate management [64]. The ketogenic diet (KD), MAD, and VLCKD have been linked to variations in MIG frequency, and elevated ketone body levels have been observed in relation to MIG symptom modulation and possible prevention of MIG episodes [43]. Some reviews [42,65] support the use of the KD as an effective preventive strategy for MIG, highlighting its role in modulating neuroinflammation, restoring metabolic balance, and reducing cortical excitability. Studies by Di Lorenzo et al. further demonstrated a significant reduction in MIG episode duration in patients undergoing ketogenic therapy [66,67]. Other trials, including those by Di Lorenzo and Bongiovanni, confirmed reductions in both the frequency and intensity of attacks [43,66,67]. Furthermore, a study comparing VLCKD with a balanced low-calorie diet (LCD) found that the ketogenic approach was more effective in reducing MIG symptoms [68].

Interestingly, a 2024 review [39] reported no significant differences between the LCD and KD with respect to mean headache frequency, supporting that both dietary approaches may offer similar benefits for some patients.

**Table 1:** Summary of the most important study concerning nutritional strategies in MIG people.

Study Design	Authors	Population	Intervention Modalities and Duration	Outcome Measures and Measurement Tools	Results
RCT	Evcili et al., 2018 [8]	294,350 MIG patients > 18 years old	3 months 147 patients: Low glycemic index diet 147 patients: pharmacological prophylaxis	Frequency and severity of attacks (using VAS)	Number of attacks per month: from 7.49 to 3.42 in diet group; from 7.53 to 2.74 in medication group VAS: from 8.46 to 1.23 in diet group; from 8.47 to 1.18 in medication group.
**Cross-sectional study**	Arab et al., 2021 [9]	262 MIG patients (20–50 years old)	10 weeks previous year: 168-item FFQ to assess the dietary intakes of participants.	Mediterranean diet score headache severity, duration and frequency MHIS HIT-6	Headache frequency (β = −1.74, 95% CI: −3.53, 0.03) Headache duration (β = −0.28, 95% CI: −0.59, −0.02) MHIS (β = −29.32, 95% CI: −51.22, −7.42) HIT-6 score (β = −2.86, 95% CI: −5.40, −0.32)
Unblinded longitudinal interventional study	Altamura et al., 2020 [50]	240 MIG patients > 18 years old	T-12 = screening T0 = educational intervention about the HEP T12 = follow-up	Anthropometric Dietary patterns (FFQ) Disability scales (MIDAS, MIDAS A, MIDAS B) HEP score MMDs Add-on to pharmaceutical therapy	BMI (−0.06) MIDAS A (T-12 = 18/T0 = 18/T12 = 15) HEP scores (T-12 = 3.5/T0 = 4.6/T12 = 4.33) Preventive therapies continued at the same dose until T12 in 74.6% of patients
RCT	Arab et al., 2022 [62]	102 women (20–50 years old)	12-week 51 women: DASH diet 51 women: usual dietary advice	24-h food record VAS questionnaire Quality of life (HIT-6) Depression, Anxiety, and Stress Scales-21 questionnaire	Dash diet vs. control: Frequency (attacks/months) = −3.00 vs. –1.4 Duration (day/attack) = −0.58 vs. –0.33 Severity = −1.76 vs. –0.59 HIT-6 scores = −3.62 vs. –2.69 Score depression = −4.5 vs. 2.73 Anxiety score = −2.74 vs. –1.46
Randomized Double-Blind, Cross-Over Trial	Di Lorenzo et al., 2019 [66]	35 episodic MIG patients (18–65 years old)	T0 = 4-week screening (non-VLCD) T1 = 4-week nutritional intervention (VLCKD or VLCnKD) T2 = 4-week progressive return to a non-VLCD T3 = control visit at the end of the 2-week of “T1” T4 = control visit at the end of the 2-week of “T2”	Mean number of MIG days per month Mean number of MIG attacks per month Mean number of doses of acute medication monthly The 50% responder rate BMI	During the VLCKD patients experienced −3.73 MIG days and −3.02 attacks respect to VLCnKD. During the VLCKD phase, 74.28% of patients achieved a ≥50% reduction in monthly MIG days, compared to only 8.57% during the VLCnKD phase. No significant differences were observed between the two dietary interventions in terms of changes in acute anti-MIG medication use or BMI
RCT	Bond et al., 2017 [69]	110 women with MIG (18–50 years old)	16 weeks 54 women: BWL (EXE and diet) intervention 56 women: ME Follow up (32–36 weeks)	MIG headache days and severity (web-based headache diary APP) Headache disability HIT-6 Anthropometric characteristics	MIG days/months: BWL group –3 (4 weeks) and –3.8 (follow up) vs. ME group –4 (4 weeks) and –4.4 (follow-up) Pain intensity: BWL group –0.8 (4 weeks) and –1.5 (follow up) vs. ME group –1.0 (4 weeks) and –0.7 (follow-up) Attack duration (h): BWL group –1.6 (4 weeks) and –2.7 (follow up) vs. ME group –5 (4 weeks) and –2.2 (follow-up) HIT-6 score: BWL group –5.4 (4 weeks) and –5.7 (follow up) vs. ME group –4.4 (4 weeks) and –5.6 (follow-up) Weight loss (Kg): BWL group –3.8 (4 weeks) and –3.2 (follow up) vs. ME group +09 (4 weeks) and +1.1 (follow-up)
**RCT**	Evans et al., 2019 [70]	110 women with MIG (18–50 years old)	16 weeks 54 women: BWL (EXE and diet) intervention 56 women: ME Follow up (16–20 weeks)	MIG headache days and severity (web-based headache diary APP) Total energy intake Percent energy from fat Diet quality (total HEI-2010 scores) Weight loss	RCT

### 3.3. Supplements and MIG

The intake of certain nutrients, such as, for example, vitamin D3, omega-3 fatty acids, coenzyme Q10 (CoQ10), magnesium, riboflavin, and alpha-lipoic acid, may play a supportive role in maintaining mitochondrial homeostasis (Table 2). Given that mitochondrial dysfunction is considered an important aspect of MIG pathogenesis—particularly in relation to brain energy deficits and oxidative stress—some studies have hypothesized that these nutrients could offer potential benefits in reducing the onset of MIG and alleviating the intensity of attacks [25,71].

#### 3.3.1. Vitamin D3

The anti-inflammatory properties of vitamin D3 (VD3) may be essential in decreasing the frequency, severity, and number of MIG days [6,71]. In their studies, some authors observed that vitamin D3 supplementation has been shown to decrease inflammatory markers and the production of nitric oxide, a key biological agent that impacts neural communication and vascular relaxation [72]. Additionally, vitamin D3 would seem to influence the secretion of serotonin and dopamine, both of which are implicated in the pathogenesis of MIG. Vitamin D3 receptors (VDRs) located in the brain could underlie the association between this micronutrient and headache; polymorphisms in these receptors may increase susceptibility to various inflammation-related conditions. A recent review [72] reported that serum vitamin D3 levels were markedly lower in MIG compared to healthy controls. Supplementation with vitamin D3, at doses ranging from 1000 to 4000 IU per day, has been shown to reduce the frequency of MIG attacks in humans with a depletion [72,73].

#### 3.3.2. Omega-3 (or PUFA *n*-3)

Some studies reported that polyunsaturated fatty acids (PUFAs), for instance docosahexaenoic acid (DHA) and eicosapentaenoic acid (EPA), may have a part in brain processes related to neuroinflammation, mitochondrial activity, pain signaling, mood regulation, and ROS imbalance [36]. In more studies, t are stated that the proportion of omega-6 (for example, Arachidonic Acid) and omega-3 modulates the inflammatory state related to the production of prostaglandins and nitric oxide (following platelet aggregation for the release of serotonin); these compounds seem to play a role in trigger pain in MIG patients [35,36,48]. A meta-analysis reveals that a fortification with 180 mg of EPA and 120 mg of DHA (administered in 6 pills for 18 weeks) can significantly reduce the frequency and severity of MIG [25,35]. Soveyd et al. [74] show that a mixture of nano curcumin (80 mg/die) and PUFA *n*-3 (2500 mg/die) minimized the transcriptional activity of TNF-α and its serum concentration.

#### 3.3.3. Coenzyme Q10

Some authors have studied the role of CoQ10, also known as ubiquinone, in various cellular redox reactions, including the electron transport chain (ETC), which is indispensable for bioenergetics and antioxidant defense. CoQ10 could help inhibit lipid peroxidation and regenerate vitamin E in its bioactive form. An 8-week supplementation regimen with nano-curcumin and CoQ10 showed some positive effects in patients with MIG, including improvements in the occurrence rate, pain level, and duration of episodes. Similarly, a daily intake of 400 mg of ubiquinone [71,75] seemed to offer some benefits in these areas, although the number of MIG days within a month does not seem to change significantly [71].

#### 3.3.4. Magnesium

Magnesium is a key cofactor in a wide range of enzymatic-mediated reactions, comprising those related to energy production, and an insufficiency in circulating levels can lead to increased oxidative stress [25,76]. It inhibits calcium channels by blocking N-methyl-D-aspartate (NMDA) receptors in neurons. These receptors may facilitate CSD and are considered potential targets for anti-MIG treatments. By blocking calcium channels in neurons, magnesium may help inhibit intracellular pro-inflammatory signaling pathways associated with MIG pathogenesis [25,76]. A state of hypomagnesemia appears to increase the risk of MIG onset [77]. Additionally, magnesium has been shown to bring down the amount of CGRP, which is targeted by recent anti-MIG drugs [77]. Supplementation with magnesium (121.5 to 600 mg per day) appears to be effective in lowering MIG frequency, severity, and the number of MIG days/month. However, it did not significantly affect attack duration [71]. Other studies have shown favorable outcomes in relation to reducing the incidence and intensity of MIG episodes, particularly with the use of organic magnesium salts. However, magnesium oxide has also been shown to be effective in preventing MIG, with similar efficacy to valproate, but without remarkable side effects, after 8 weeks [78].

#### 3.3.5. Riboflavin

Supplementing with Riboflavin appears to substantially lower the rate of occurrence and severity of MIG, primarily due to its crucial role in myelin synthesis. Riboflavin is an essential micronutrient for maintaining mitochondrial equilibrium, producing energy, and protecting the brain in defense against ROS [25]. The guidelines of the American Academy of Neurology suggested that riboflavin should be considered a Level B option for preventing MIG, with strong evidence supporting its benefit. Daily intake of 400 mg for three months may help reduce the frequency of MIG episodes, with fewer adverse effects compared to drug treatments [79]. On the other hand, there does not appear to be a correlation between an adequate intake of dietary riboflavin and the risk of MIG [80].

#### 3.3.6. Alpha-Lipoic Acid

Alpha-Lipoic Acid (α-LA) is a compound known for its potential to inhibit oxidation. It helps in replenishing other antioxidant molecules and may contribute to healthier mitochondrial functions. This includes potentially enhancing the activity of mitochondrial superoxide dismutase, which is important for cellular health [25]. Gross et al. observed that 85% of patients with high-frequency MIG had lower serum levels of *α-LA*. Daily doses of 300 to 600 mg have proven effective in attenuating both the frequency and intensity of MIG [71]. According to Rezaei et al. [81], 600 mg/day of α-LA (for 3 months) affects mitochondrial and endothelial function, helping to control oxidative stress and inflammation in those affected by MIG [82].

#### 3.3.7. Folates

The use of folates is receiving increasing attention; they are involved in DNA methylation and homocysteine metabolism, with potential beneficial effects in MIG management. Low folate concentrations in the blood are associated with an elevated likelihood of developing MIG [37,83].

#### 3.3.8. Probiotics

Probiotics supplementation may improve quality of life by reducing MIG attacks by various mechanisms, including the formation of SCFAs (short-chain fatty acids), enhancement of intestinal barrier integrity, reduction in pro-inflammatory compounds, and alleviation of gastric stasis [84]. However, some studies have reported conflicting results, and further investigations are needed to validate and elucidate these outcomes in the context of multiple supplementation products.

**Table 2:** Summary of the most important study concerning supplements in MIG people.

Study Design	Authors	Population	Intervention Modalities and Duration	Outcome Measures and Measurement Tools	Results
RCT	Ramsden et al., 2021 [34]	182 MIG patients (mean age 38 years)	16 weeks H3 diet = EPA + DHA to 1.5 g/day and linoleic acid at 7% of energy H3-L6 diet = EPA + DHA to 1.5 g/day and linoleic acid to ≤1.8% of energy Control = EPA + DHA at <150 mg/day and linoleic acid at 7% of energy.	17-HDHA in blood Daily Headache frequency (electronic diary) HIT-6	The H3-L6 and H3 diets increased circulating 17-HDHA Total headache hours per day: −1.3 (H3) and –1.7 (H3–L6) HIT-6 score= −1.5 (H3) and –1.6 (H3–L6) The H3-L6 diet decreased headache days per month more than the H3 diet. The H3-L6 and H3 interventions did not significantly improve quality of life.
RCT	Gazerani et al., 2019 [73]	48 MIG patients (18–65 years of age)	24 weeks 24 patients: 100 μg/day D3-Vitamin 24 patients: placebo	MIG attack frequency, number of days and severity (self-reported diaries) Mig-related symptoms Quantitative sensory tests HIT-6 25(OH)D and 1.25(OH)2D serum levels	Attack frequency: from 3.00 to 1.29 (D3) Attack severity: from 2.16 to 1.87 (D3) Number of days with MIG: from 6.25 to 3.28 (D3) No significant changes were observed for symptoms, PPT and temporal summation HIT-6 score: from 63.25 to 53 (D3 group) 25(OH)D levels increased significantly for the D3-Vitamin group during the first 12 weeks of treatment
RCT	Dahri et al., 2017 [75]	84 women with MIG (18–50 years)	12 weeks 42 women: 400 mg/day CoQ10 42 women: placebo (P) (both with usual prophylactic drugs a month before)	MIG frequency/month MIG severity (VAS) MIG duration (hour) HIT-6 MIDAS MSQ	From 8.20 to 3.55 (Q10) and 6.47 to 3.76 (P) From 8.35 to 4.46 (Q10) and 7.11 to 4.97 (P) From 11.98 to 4.79 (Q10) and 10.80 to 6.72 (P) HIT-6 Score −12.51 (Q10) and –8.74 (P) MIDAS Score –16.39 (Q10) and –8.24 (P) MSQ role restrictive +39.78 (Q10) and +16.54 (P) MSQ role preventive +33.46 (Q10) and +11.31 (P) MSQ emotional functioning +35.21 (Q10) and +16.49 (P) The most significant effect of CoQ10 was a reduction in the duration of attacks.
RCT	Karimi et al., 2021 [78]	70 MIG patients (18–65 years)	2 sequences of 8 weeks G1: 500 mg magnesium oxide and then 400 mg valproate Na G2: 400 mg valproate Na and then 500 mg magnesium oxide (two tablets/day)	VAS HIT-6 Scores MIDAS scale Duration (h) MIG attacks/MONTHS number of MIG days	Both treatments resulted in a significant reduction in the frequency, duration, and intensity of MIG attacks and associated symptoms compared to baseline values, without showing statistically significant differences.
RCT	Rahimdel et al., 2015 [79]	90 MIG patients (15–55 years)	3 months G1: vitamin B2 treatments of 400 mg/day G2: 500 mg/day of Na valproate	Duration of MIG pain Frequency of MIG episodes Headache severity (VAS)	From about 15.1 ± 7.1 to 4.2 ± 2.6 hr/month (G1) and from 16.2 ± 10.6 to 8.2 ± 4.7 hr/month (G2) From 9.2 ± 6.2 to 2.4 ± 1.6 times/month (G1) and from 6.5 ± 3.1 to 2.1 ± 1 times/month (G2) Score decreased in 71.8% of G1 and 76.2% of G2 Both treatments were similarly effective, but vitamin B2 caused fewer side effects
RCT	Kelishadi et al., 2022 [82]	92 women with MIG (20–50 years)	12 weeks G1 (300 mg/day ALA) and G2 (placebo) twice per day	Headache severity (VAS) Frequency/month Duration of attacks HDR HIT-6 MHIS	Mean change: −3.59 (G1) and −0.70 (G2) Mean change: −2.55 (G1) and −0.40 (G2) Mean change: −19.49 (G1) and −15.37 (G2) Mean change: −158.79 (G1) and −38.63 (G2) Mean change: −20.09 (G1) and −2.83 (G2) Mean change: −65.32 (G1) and −0.33 (G2) The result on duration was similar in both.

### 3.4. Relationship Between PA and MIG

There is moderate evidence of a curvilinear relationship, similar to the dose–response association, between the volume of PA and specific health effects, such as a decrease in mortality, cardiovascular disease, and the incidence of Non-Communicable Diseases, such as cancer and diabetes [85]. The health benefits already occur with levels of PA lower than recommended, which supports the WHO’s claim that practicing some PA is better than not practicing it at all.

Many authors recommend maintaining an active lifestyle [86] for people with MIG. Oliveira AB et al. (2021) showed that lower-than-guideline levels of PA are related to the central primary headache disorders, particularly MIG [87]. In a cross-sectional study conducted by Denche-Zamorano et al. [88], researchers examined the link between MIG and PA levels in adults of Spain. They also explored how levels of PA relate to depression, anxiety, and self-perceived health among individuals with MIG. The findings indicated that MIG prevalence is associated with PA levels. The group with very high levels of PA demonstrated the lowest frequency of MIG (7.5%) in contrast to the inactive group, which had a higher prevalence (13.3%). Additionally, physically inactive individuals with MIG showed a higher prevalence of depression (36.6%) and anxiety (33.2%), as well as a more negative perception of their health compared to those who were physically active. Leisure-time PA (LTPA) levels are negatively correlated with the frequency of headache attacks and the occurrence and prevalence of MIG [89,90]. Moreover, it is noted that daily step counts are inversely related to the incidence of MIG [91].

In a separate study, Hagen et al. [92] found that patients with MIG had a low average peak oxygen uptake (VO_2_ peak). After a 12-week EXE intervention, patients showed an increase in VO_2_ Peak, but no change in MIG frequency was reported.

Some individuals who experience MIG have identified EXE as a potential trigger for their attacks or as a factor that can intensify pain during an episode [93]. This may contribute to a tendency among MIG sufferers to limit their PA. It is essential to acknowledge that scientific literature presents a limited number of studies demonstrating that EXE induces MIG attacks. In an observational study, participants reported that vigorous and moderate-intensity physical activity were 61% and 51% likely, respectively, to trigger an MIG attack. They also believed that such activities worsened MIG pain, with an 84% likelihood for vigorous activities and a 75% likelihood for moderate activities. Experiencing MIG triggered or aggravated by PA may shape beliefs about its adverse effects, leading individuals to avoid it [94]. Farris et al. 2019 [95] examined the intentional avoidance of PA in women with MIG and its relationship to their leisure PA levels and observed that a significant portion of participants, accounting for 78%, reported often avoiding PA (mean = 4 days/week) to prevent the possibility that PA, particularly at a higher intensity, might trigger or potentially intensify an MIG attack [95]. Participants who avoided PA had twice as many MIG attacks in the past month compared to those who did not. Furthermore, more frequent avoidance was associated with a greater number and longer duration of attacks [94].

### 3.5. Overview of Different PA Approaches Explored in MIG

Among non-pharmacological treatments, regular physical EXE (a specific type of PA defined as planned, structured, repetitive, and aimed at improving or maintaining physical fitness [96]) is frequently suggested as a way to alleviate MIG symptoms and possibly linked pathologies.

The effects of physical EXE have been studied, and several clinical studies, with protocols involving different types of EXE, have demonstrated its effectiveness in managing MIG (Table 3). Nonetheless, there hasn’t been a direct comparison of the effectiveness of various EXE interventions [97]. Strength/resistance training produces the most significant benefits in reducing MIG frequency. This effect appears to be due to targeted muscle strengthening and reconditioning, which particularly involves the main muscles of the neck, shoulders, and upper limbs. Although the evidence remains preliminary, the potential mechanisms behind the therapeutic neck strength EXEs may involve local metabolic and neuromuscular adaptations, along with an increase in relative strength [97]. In relation to aerobic exercise (AEREXE), the enhanced benefits of high-intensity AEREXE might be related to the intensity-specific activation of endogenous molecules that seem to play a role in exercise-induced hypoalgesia [98]. Some author reported that this dose-dependent effect of AEREXE on MIG management could be linked to improvements in mitochondrial and cardiorespiratory functions [99] and anti-inflammatory mechanisms associated with AEREXE [100]. Additionally, a paper by Kroll et al. confirmed the importance of regular AEREXE in improving the burden of MIG [101].

Although some headache societies [19,20,21] include EXE in the multidisciplinary treatment of MIG, univocal indications for EXE prescription are not well elucidated. However, some authors attempt to provide recommendations for healthcare and EXE professionals [102] (Table 4).

In 2023, La Touche et al. [102] synthesized recommendations based on the best available evidence. They assigned grade B of recommendation to general (not defined for prescription) AEREXE. AEREXE is efficacious in ameliorating the number of attacks, intensity, and duration of MIG in people with MIG, thereby improving their quality of life. Notably, at continuous AEREXE played at moderate intensity (American College of Sports Medicine, or ACSM, defined as “moderate” a PA played at 40–59% of the heart rate reserve, or the oxygen uptake reserve) was assigned a B grade of recommendation. Finally, yoga was found to have a B-grade recommendation for reducing headache frequency and disability. Other practices, such as continuous AEREXE played at low intensity (ACSM defined as “low” a PA played at 20–39% of the heart rate reserve, or the oxygen uptake reserve), relaxation EXEs, and high (ACSM defined as “high” a PA played at 60–84% of the heart rate reserve, or the oxygen uptake reserve)-intensity interval training received a recommendation grade of Cin improving headache frequency.

Several clinical studies have analyzed the effectiveness of various types of physical EXE in reducing the frequency, intensity, duration, and disability of MIG patients. In 2022, Woldeamanuel & Oliveira [97] conducted a systematic review and meta-analysis of clinical trials that assessed the effectiveness of EXE interventions in reducing the monthly frequency of MIGs. The review compared the efficacy of moderate-intensity AEREXE, high-intensity AEREXE, and strength/resistance training. It found that strength training was most successful in decreasing MIG burden, followed by high-intensity AEREXE. Strength training may excel due to its ability to strengthen muscles, particularly in the neck, shoulders, and upper extremities, linked to the trigeminal-cervical system associated with MIG. High-intensity AEREXE offers additional benefits by activating endogenous molecules involved in EXE-mediated hypoalgesia, particularly within the opioid and endocannabinoid systems, which are often deficient in MIG patients. Research indicates that endorphin secretion and cerebral opioid receptor binding are more significant during high-intensity EXE than moderate-intensity EXE [97].

Although the evidence remains preliminary, in another work by Reina-Varona et al. (2023) [103], experts recommend moderate-intensity continuous aerobic EXE as the most effective approach for alleviating MIG symptoms. Each session should begin at 30 min and gradually extend to a minimum of 60 min over time, with a frequency of three days a week. At least eight weeks of this EXE is needed to see improvements in MIG pain. Additionally, relaxation and breathing EXEs should be done daily for 5 to 20 min, as they effectively reduce MIG frequency [103].

Although the evidence is still not definite, in a systematic review by Reina-Varona Á et al. (2024) [104], involving 28 studies with 1501 participants, it was found that yoga, high-intensity AEREXE, and continuous moderate-intensity aerobic EXE were more effective than drug therapy alone for reducing MIG frequency. Yoga was especially effective in decreasing the intensity of attacks, while high-intensity AEREXE significantly reduced their duration, and moderate-intensity EXE helped lessen associated disability. Krøll et al. [101] emphasized that aerobic endurance training could have a positive impact on the frequency, intensity, and duration of MIG episodes, thereby enhancing overall patient well-being.

### 3.6. Sedentariness and Avoidance of PA

To the best of our knowledge, the actual evidence examined on sedentary behavior and health outcomes in adults supported the idea that all adults should limit the amount of time spent sedentary [85]. Data on leisure PA levels seem to suggest that physical inactivity may be linked to an increased risk of developing significant primary headache disorders. Additionally, engaging in regular physical activity may be beneficial in managing and potentially reducing the chronic development of these headache conditions.

Moreover, physical inactivity is associated with psychological disorders and symptoms like anxiety, with MIG being a significant factor [105].

On the other hand, it’s important to underline that regular headache pain could represent a barrier to PA in people’s free time. Bond et al. (2015) [69], monitoring daily PA through ambulatory methods, stated that people with MIG have lower PA levels than the normal population. Rogers et al. (2020) [106] confirmed that people with MIG made less PA than people without MIG. Moreover, the PA level did not differ significantly between MIG days and non-MIG days. Recent evidence shows the involvement of physiological and psychological mechanisms underlying MIG attacks triggered by PA and its avoidance. Particularly, the intentional avoidance of movement, due to the fear of pain aggravation by movement, was defined as kinesiophobia [107]. Kinesiophobia, or the fear of movement, affects approximately 53% of individuals who suffer from MIG. Additionally, a study by Benatto et al. found that nearly 70% of participants believed that physical activity should be avoided due to concerns about the risk of injury and the potential worsening of their headaches. Furthermore, more than 50% of those experiencing kinesiophobia reported a fear of injuries related to EXE [107].

### 3.7. Weight Loss Strategies: Obesity and MIG

MIG and obesity are two chronic disorders that share standard pathophysiological mechanisms, which may influence each other [108]. Several hypothetical mechanisms underlying this interplay involve a chronic inflammatory state characterized by the release of biochemical markers, including neuropeptides, pro-inflammatory mediators, and adipokines [17,109]. These factors are linked to the release of CGRP from trigeminal afferents, whose sensitization increases susceptibility to headaches in individuals with obesity. Hypothalamic neuropeptides, including neuropeptide Y and orexin, regulate both energy intake and expenditure, while leptin and adiponectin, secreted by adipocytes, are involved in energy homeostasis and inflammatory processes [109]. Adipokines, in particular, elevated leptin levels, are associated with inflammatory processes through the release of pro-inflammatory cytokines, which are involved in both MIG attacks and obesity [109]. Additionally, leptin is linked to CSD, suggesting that increased leptin levels in obesity may trigger chronic MIG. Razeghi Jahromi et al. [41] indicated that elevated adiponectin levels may have a beneficial effect on MIG reduction, as adiponectin may block the production of TNF-α and IL-6. In obese individuals, a state of chronic inflammation exacerbates the frequency, severity, and duration of MIG attacks. In fact, fat intake in obesity may promote the release of CGRP by increasing sensory nerve activity, which in turn raises substance P levels. These compounds contribute to arterial vasodilation, enhanced vascular permeability, and mast cell degranulation [110]. Some authors have explored the role of obesity as a modifiable risk factor that can worsen MIG [111]. They suggested that losing weight could be a beneficial treatment approach for those dealing with both conditions. Interventions for weight loss may encompass behavioral weight loss strategies, medication, and/or bariatric surgery, depending on how severe the obesity is [111]. BWL incorporates behavioral therapy alongside diet and physical activity interventions, promoting lifestyle changes that facilitate weight reduction [17,111,112]. Dietary interventions and physical activity can help control body weight, which in turn influences inflammation, hormones, sleep, and breathing, all of which may impact headache frequency and severity. Weight loss has been shown to improve MIG characteristics in obese patients, regardless of the type of intervention or the extent of weight loss [113]. A mean reduction in BMI of 4.1 kg/m^2^ led to a significant decrease in MIG frequency, pain severity, headache disability, and a small but significant reduction in headache duration [113]. These findings can be attributed to the fact that energy restriction reduces the production of pro-inflammatory cytokines. Some studies [114,115] hypothesized that individuals with obesity could have a greater risk of MIG, with geographic variation contributing to this difference. Moreover, it seems that the buildup of visceral fat—that is often seen as a characteristic of obesity- could be linked to increased rates of MIG [115]. The incidence of MIG was observed to rise in parallel with an increase in the waist-to-height ratio in adults up to 60 years [116]. Furthermore, a meta-analysis [108] revealed a nonlinear association, indicating an increased risk of MIG in both underweight and obese individuals. Specifically, the risk of MIG increased by 12% at a BMI of 17, and by 6%, 26%, and 51% at BMI values of 30, 35, and 40, respectively. However, the study by Evans WE et al. found that a BWL intervention in women with MIG and a BMI greater than 25 was not associated with significant improvements in either MIG outcomes or weight reduction [70]. Finally, here is some evidence [117] suggesting that obesity could be linked to MIG, with various factors like neuropeptides, inflammatory mediators, adipokines, gut microbiota, and changes in eating habits and lifestyle may play a role. To better understand the relationship between MIG and obesity, further research involving comprehensive prospective studies is needed.

## 4. Discussion

According to current literature, lifestyle factors play a key role in the pathogenesis, prevention, and management of MIG. An interdisciplinary approach that integrates dietary modifications plus tailored physical activity, aimed at reducing systemic inflammation and, in cases of obesity, promoting weight loss, may represent an effective therapeutic strategy.

A balanced, healthy, anti-inflammatory, and low-glycemic index diet approach, following the Mediterranean model, could be more sustainable over time and may have positive effects on MIG control through the alleviation of the inflammatory state [41]. Therefore, the choice of unrefined plant foods such as whole grains, fruits, vegetables, legumes, nuts, and seeds will be the focus of this ideal approach, as well as an adequate consumption of fish (especially oily) and eggs, limiting dairy products, refined carbohydrates, red meat, and Na (like DASH diet [62]). The same approach can be used in the case of an obese patient with MIG, but considering weight loss as a key element for success. Numerous benefits can be obtained by preventing or improving MIG symptoms through the use of vitamin D3 (if the patient is deficient), omega-3 fatty acids (EPA/DHA), magnesium, CoQ10, riboflavin, and alpha-linolenic acid. Attention should also be paid to the proper hydration and the moderate consumption of alcohol, caffeine, and MSG. Despite growing evidence supporting the efficacy of ketogenic interventions, KD is still not routinely recommended as a therapeutic option in most clinical guidelines for chronic MIG. The nutritional approach should be adapted to individual pathophysiological needs and then fine-tuned with specific nutrients for each case. Several nutrients can be supplemented to prevent and/or improve MIG by modulating oxidative stress. Each person may react differently to foods, so creating a food diary [118] to identify foods that trigger or relieve MIGs may be helpful.

In addition to a healthy diet approach, an increase in PA level and a reduction in sedentary time are the main objectives in people with MIG. Despite the health effects of PA being well established, people with MIG PA levels are lower than those of the general population [88]. For these reasons, it is crucial to propose lifestyle interdisciplinary interventions that allow people with MIG to be more active. Although our previous study focused on the barriers to a healthy lifestyle in patients with chronic Non-Communicable Diseases [119], it is necessary to underline that in people with MIG, the barriers are quite different. For instance, among the most frequent obstacles, people with MIG experience the fear of triggering or worsening an MIG episode [120], avoidance behaviors, and kinesiophobia [107]. For these aspects, although the evidence is preliminary, some authors suggested that new strategies, such as more flexible tele-coaching training modality [93,104,121], interdisciplinary and PA behavioral counseling, or integration with headache education programs [122], could help overcome these barriers. Moreover, understanding the barriers and beliefs of people with MIG, it is crucial to implement PA and prescribe the optimal EXE program (EP). Regarding the contents of the EP, according to the acronyms F.I.T.T. [123], which identify frequency, intensity, typology, and time (duration) as defined by the ACSM, they could be critical to determine the weekly recommended EXE. Several studies have attempted to explain the varying effects of different PA typologies [97,99,101,104]. Strength EXE seems to be more effective in reducing MIG frequency. At the same time, aerobic exercise plays an anti-inflammatory role, and it has a primary impact on the intensity and duration of MIG attacks. Notably, about EXE intensity, Woldeamanuel and Oliveira [97] showed that high-intensity aerobic EXE and moderate-intensity continuous aerobic EXE were significantly superior to drug treatment alone in reducing MIG frequency. For reducing MIG duration, high-intensity aerobic exercise and moderate-intensity continuous aerobic exercise were superior to drug treatment alone. Moreover, yoga is effective (B-grade recommendation) in reducing headache frequency and disability [104], as well as duration and intensity [97]. Finally, regarding leisure-time activities, such as walking, it is noted that daily step counts are linked to a better response to anti-CGRP monoclonal antibody treatment [124].

## 5. Conclusions

Although evidence remains preliminary, to manage MIG, it seems to be crucial to have an interdisciplinary approach that includes dietary changes, regular PA, and weight management.

A balanced, anti-inflammatory diet based on the Mediterranean model (rich in unrefined plant foods, whole grains, fruits, vegetables, nuts, seeds, and fish, while limiting refined carbohydrates and Na) has shown potential in controlling MIG.

Moreover, supplements such as vitamin D3, omega-3 fatty acids, magnesium, CoQ10, riboflavin, and alpha-lipoic acid may help to alleviate inflammation and oxidative stress associated with MIG. Given the importance of the link between Mig and DIET, supported by the literature reported in this paper, it is useful to continue investigations.

PA plays a crucial role in MIG management, according to some studies that have explored the benefits of some type of EXE, such as strength training and high-intensity aerobic exercise. It’s important to address barriers to PA, such as fear of triggering attacks, through personalized exercise programs and education.

For individuals with both obesity and MIG, weight loss interventions could improve outcomes. Given the bidirectional relationship between obesity and MIG, addressing both conditions together seems to be vital.

An interdisciplinary approach that combines nutrition, PA, and weight management could offer the most significant potential for effective MIG prevention and treatment. Personalized strategies that consider individual triggers have been reported to as important for achieving optimal results in the adult population. Future studies should aim to enhance these strategies and assess their lasting effectiveness among MIG and various populations.

## Figures and Tables

**Table 3:** Summary of the most important study concerning physical activity aspects in MIG people.

Study Design	Authors	Population	Intervention Modalities and Duration	Outcome Measures and Measurement Tools	Results
Prospective multicenter cohort study	Oliveira AB et al. (2021) [87]	15,105 civil servants (35 and 74 years)	Workplace-based interviews and clinic visits for biochemical sampling and assessments.	Pain frequency, duration, quality, location, intensity, triggering factors, and accompanying symptoms. Commuting (CPA) and leisure-time (LTPA) physical activity. Questionnaires were used.	Regression models for the LTPA domain showed increased odds ratio (95% CI) for MIG (OR: 1.37 [1.16–1.61], *p* < 0.001 in the “inactive” level. MIG subtypes were also associated with “somewhat active” (OR: 1.27 [1.02–1.56], *p* < 0.05)
Cross-sectional study	Denche-Zamorano et al. (2022) [88]	17,139 of which 1972 MIG urs (18 and 70 years)	Interviewers between October 2016 and October 2017.	Physical activity level (PAL). Self-perceived health (SPH). Questionnaires were used.	MIG prevalence was lowest among individuals with a very active physical activity level (7.5%), and only 49.8% of those with MIG reported a positive perception of their health.
Population-based historical cohort study	Hagen et al. (2018) [89]	15,276 participants without headache	Survey in the HUNT population of 1995–1997 and 2006–2008.	Relationship between baseline lifestyle factors and risk of headaches 11 years later.	Hard physical EXE for 1 to 2 h per week reduces MIG risk (OR 0.71, 95% CI 0.54–0.94), while smokers have a higher risk (RR 1.30, 95% CI 1.11–1.52).
RCT	Hanssen et al. (2017) [99]	48 people with MIG	three groups: high intensity aerobic interval training group (HIT); moderate continuous aerobic training group (MCT); control group (CON). Twice a week, 12 weeks.	Effect of exercise training on pulse wave reflection. Arterial Stiffness, using an oscillometric device; Migraine Days; VO_2_ max	Stiffness :moderate effects in HIT [pre: 22.0 (9.7), post: 14.9 (13.0), SMD = 0.62]; MIG Days: very large effects in HIT [pre: 3.8 (3.0), post: 1.4 (1.2), SMD = 1.05]. VO_2_ max_:_ moderate effects in favor of HIT [(ml/min/kg) pre: 36.8 (5.2), post: 41.3 (8.3), SMD = −0.65]
RCT	Krøll et al. (2018) [101]	70 with MIG, tension-type headache, neck pain.	Diagnosed by a neurologist plus physical examination by an experienced physiotherapist. Two groups: EXE group and control group. From January 2013 to July 2015.	Headache characteristics with a diary. Level of physical activity (IPAQ), Well-being (WHO-5), MIG impact (Impact M-TTH-NP), through questionnaires. VO_2_ max, using Åstrand’s submaximal bicycle test.	Significant reduction was found in the EXE group for MIG frequency (31%; *p* = 0.19), pain intensity (*p* = 0.005), and duration (*p* = 0.045). The EXE group significantly improved their physical fitness (*p* = 0.014) and well-being (*p* < 0.001).

**Table 4:** EXE evidence for MIG people.

Authors and Study Design	Type of EXE	Intensity of EXE	Duration of EXE	Frequency/Week of EXE	Results
Woldeamanuel & Oliveira [97], systematic review	Strength/resistance training	From 45–60% to 75–80% of 1 RM	From 2–3 sets of 12–15 repetitions to 3 sets of 8–10 repetitions	Thrice/week, 45–60 min per session, 8–12 weeks.	Monthly MIG days = −3.55 [− 6.15, − 0.95]).
La Touche et al. [102], Clinical practice guidelines	AEREXE, grade of recommendation B	without a specific EXE prescription parameters			Reduces the frequency, intensity, and duration of pain to enhance overall quality of life.
	continuous AEREXE, grade of recommendation B	40–59% HRR, from 12 to 16 on the Borg perceived exertion scale		Thrice/week, 8 weeks	Reduces headache frequency, may lessen pain intensity, and positively impacts attack duration, disability, and quality of life
	Yoga, grade of recommendation B	From light to vigorous	N.A.	Thrice/week, 6 weeks	Reduces the frequency and severity of headaches, while also providing remote relief for pain intensity and the duration of attacks.
	continuous AEREXE, grade of recommendation C	60–84%			Reduces the frequency
	continuous AEREXE, grade of recommendation C	20–39% HRR			Reduces the frequency
	Relaxation EXEs, grade of recommendation C			Once a week, 6 weeks Thrice/week, 12 weeks	Remotely improve headache frequency Remotely improves pain intensity
	High-intensity interval training, grade of recommendation C				May enhance pain frequency and potentially reduce pain intensity, duration, and disability.
	Yoga, grade of recommendation B	From light to vigorous	N.A.	Thrice/week, 6 weeks	Reduces the frequency and severity of headaches, while also providing remote relief for pain intensity and the duration of attacks.
	High-intensity AEREXE	From 55–60% to 80–90% VO_2_ max	45–60 min	2–3 times a week, 8–12 weeks.	Monthly MIG days = −3.13 [−5.28, −0.97]).
	moderate-intensity AEREXE	45–70% VO_2_ max	45–60 min	Thrice/week, 8–12 weeks.	Monthly MIG days = −2.18 [−3.25, −1.11]).
Reina-Varona et al. (2023) [103], systematic review	Continuous AEREXE	moderate-intensity (40–59% of HRR)	From 30′ to 60′ (at least eight weeks)	three days a week	-
	Relaxation and breathing EXEs	N.A.	5′ to 20′	every day	Reduce MIG frequency
Reina-Varona Á et al. (2024) [104], systematic review and network meta-analysis	Yoga, very-low-quality evidence	N.A.	N.A.	N.A.	Frequency:(SMD −1.30; 95% CI −2.09, −0.51). Intensity: (SMD −1.40; 95% CI −2.41, −0.39).
	AEREXE, very-low-quality evidence	high-intensity	N.A.	N.A.	Frequency: (SMD −1.30; 95% CI −2.21, −0.39). Duration: (SMD −1.64; 95% CI −2.43, −0.85)
	Continuous AEREXE, very-low-quality evidence	moderate-intensity	N.A.	N.A.	Frequency:(SMD −1.01; 95% CI −1.63, −0.39) Duration: (SMD −0.96; 95% CI −1.50, −0.41).

Legend: AEREXE = aerobic exercise; EXE = exercise; N.A. = not available; HRR = heart rate reserve; SMD = Standardized mean difference; CI = confidence interval.

## Data Availability

Data sharing is not applicable.

## Abbreviations

The following abbreviations are used in this manuscript:

MIG	Migraine
PA	Physical Activity
KD	Ketogenic diet
MD	Mediterranean diet
NH	Nutritional habits
EXE	Exercise
CGRP	Calcitonin Gene-Related Peptide
CSD	Cortical Spreading Depression
MIDAS	Migraine Disability Assessment score
MMDs	Monthly Migraine Days
BWL	Behavioral Weight Loss
ME	Migraine Education
FFQ	Food Frequency Questionnaire
17-HDHA	Antinociceptive mediator 17-Hydroxydocosahexaenoic Acid
HIT-6	Headache Impact Test
VAS	Visual Analog Scale
LCD	Low-Calorie Diet
VLCD	Very-Low-Calorie Diet
VLCKD	Very-Low-Calorie Ketogenic Diet
VLCnKD	Very-Low-Calorie non-Ketogenic Diet
MHIS	Migraine Headache Index Score
MSQ	Migraine Specific Quality of Life
